# Multiple Distant Origins for Green Sea Turtles Aggregating off Gorgona Island in the Colombian Eastern Pacific

**DOI:** 10.1371/journal.pone.0031486

**Published:** 2012-02-02

**Authors:** Diego F. Amorocho, F. Alberto Abreu-Grobois, Peter H. Dutton, Richard D. Reina

**Affiliations:** 1 School of Biological Sciences, Monash University, Clayton, Victoria, Australia; 2 Research Center for Environmental Management and Development, Cali, Colombia; 3 Unidad Académica Mazatlan, Instituto de Ciencias del Mar y Limnología, Universidad Nacional Autónoma de Mexico, Mazatlan, Sinaloa, Mexico; 4 Protected Resources Division, Southwest Fisheries Science Center, National Oceanic and Atmospheric Administration, La Jolla, California, United States of America; Institut Pluridisciplinaire Hubert Curien, France

## Abstract

Mitochondrial DNA analyses have been useful for resolving maternal lineages and migratory behavior to foraging grounds (FG) in sea turtles. However, little is known about source rookeries and haplotype composition of foraging green turtle aggregations in the southeastern Pacific. We used mitochondrial DNA control region sequences to identify the haplotype composition of 55 green turtles, *Chelonia mydas*, captured in foraging grounds of Gorgona National Park in the Colombian Pacific. Amplified fragments of the control region (457 bp) revealed the presence of seven haplotypes, with haplotype (*h*) and nucleotide (π) diversities of *h* = 0.300±0.080 and π = 0.009±0.005 respectively. The most common haplotype was CMP4 observed in 83% of individuals, followed by CMP22 (5%). The genetic composition of the Gorgona foraging population primarily comprised haplotypes that have been found at eastern Pacific rookeries including Mexico and the Galapagos, as well as haplotypes of unknown stock origin that likely originated from more distant western Pacific rookeries. Mixed stock analysis suggests that the Gorgona FG population is comprised mostly of animals from the Galapagos rookery (80%). Lagrangian drifter data showed that movement of turtles along the eastern Pacific coast and eastward from distant western and central Pacific sites was possible through passive drift. Our results highlight the importance of this protected area for conservation management of green turtles recruited from distant sites along the eastern Pacific Ocean.

## Introduction

Genetic, tagging and satellite tracking studies have demonstrated that green turtles, *Chelonia mydas*, spend an early pelagic stage in the ocean, followed by a neritic stage in which juveniles settle in coastal foraging grounds (FG) [1]–[3]. These areas can be either shared with adults (and will constitute the adult residential foraging grounds where juvenile turtles will later spend their inter-reproductive periods), or be frequented only by juveniles, that will later shift to a different adult feeding area [4]. These FG are used as developmental areas, stopover habitats and refueling stations during turtles' life cycles [5]. Sea turtle foraging aggregations commonly consist of genetically mixed stocks made up of turtles originating from different distant rookeries [6]–[9], although it was recently demonstrated that green turtle foraging populations in the central Pacific consisted of a single Hawaiian genetic stock [10]. Understanding the links between FG stocks and the breeding rookeries from which animals originate is of great importance to developing holistic conservation strategies for this trans-boundary species [10], and there is a need to pursue genetic studies to determine the stock composition at FG for green turtles in the eastern Pacific [8], [11]–[13].

**Figure 1:**
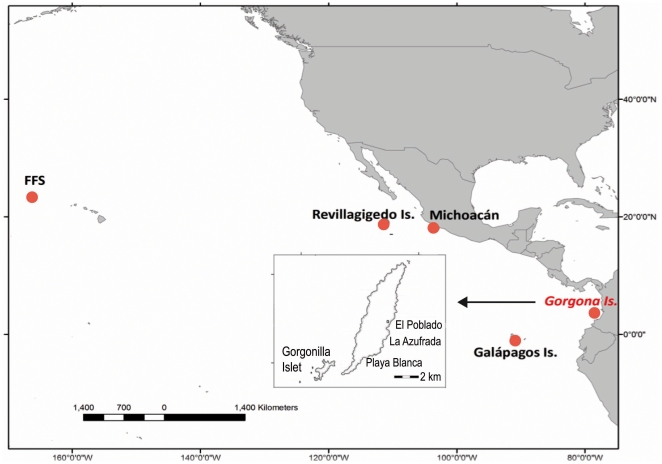
Location of Gorgona National Park in the Pacific. Dots represent locations from where haplotypes were identified, excluding an Australasian hypothetical rookery. Turtles were caught by hand in the coral reefs of La Azufrada and Playa Blanca on the east side of the island.

**Figure 2:**
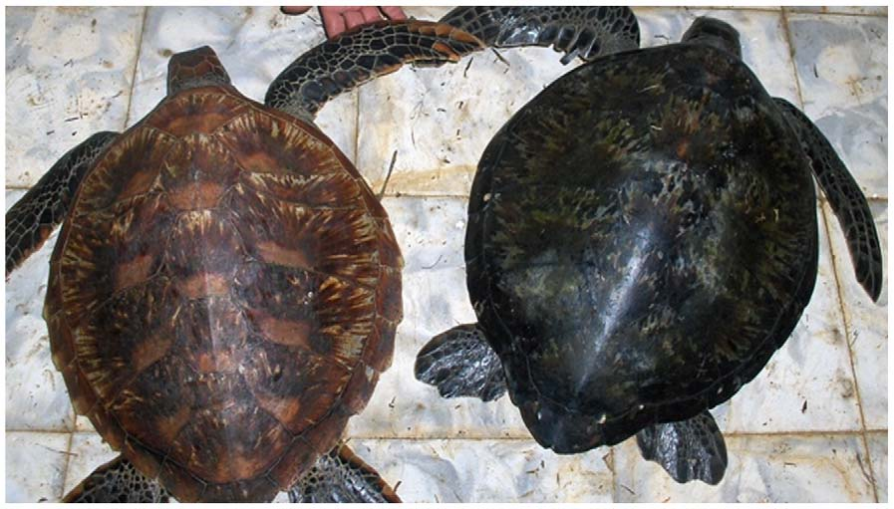
Observed variations in carapace color and shape of green turtle (*Chelonia mydas*) juveniles. Variations corresponding to the Australasian (CMP21, CMP 22 and CMP 97) and central/eastern Pacific (CMP4, CMP 8, CMP 5, CMP 17) haplotypes (left and right turtles, respectively) caught at the Gorgona foraging study site. Putative west Pacific turtles exhibited a much lighter golden-brown coloration with indentation in the lower carapace edges, in contrast to the darker “black” carapaces of the typical eastern Pacific individuals. Photo: Javier Rodríguez-Zuluaga.

Green turtles of about 45–65 cm curved carapace length (CCL) forage all year round in the Marine Protected Area (MPA) of Gorgona Island National Park in Colombia (Figure 1). The island foraging aggregation is thought to comprise transient, mostly juvenile individuals that pass through Gorgona on their way from one FG to another. This is concluded from their diet, demographic structure and the low recapture rate of animals from year to year [14], [15]. Further, the striking morphological differences in carapace shape and coloration (Figure 2), suggest that turtles converging on this small island might have their origins in multiple rookeries. In order to test this speculation and to identify the geographic regions from which turtles may have come, we characterized the genetic composition of Gorgona's FG aggregation. The mtDNA control region has been used extensively to characterize stock structure and patterns of dispersal in sea turtles [9]–[11], [16]–[19]. In this study we used mtDNA control region sequences to identify the genetic stock composition and infer the contributions from the eastern, central and western Pacific regions to the Gorgona FG aggregation. Information on the geographic origins of turtles at this FG has important conservation implications, because the significant mortality caused by illegal artisanal fleets operating around Gorgona in the Colombian Pacific [20], [21] potentially affect breeding populations on a regional geographic scale [22].

In this phase of a long-term study as part of the Gorgona National Park Sea Turtle Action Plan, our main objectives were to carry out a genetic characterization of the green turtles foraging on Gorgona waters and to estimate the contribution from Pacific-wide stocks to the island.. Knowledge from this investigation will be useful to better understand green turtle ecology, improve regional conservation management strategies and aid understanding of oceanographic currents and patterns that might play a significant role in green turtle dispersal within the Pacific Ocean.

## Materials and Methods

### Ethics statement

This study was conducted under research permit DTSO 0029 from the Unidad Administrativa Especial del Sistema de Parques Nacionales Naturales, contract for access to genetic resources issued by the Colombian Ministry of the Environment 28/03/2007 and ethics approval BSCI/2003/04 from Monash University.

### Study site and sample collection

Turtles were captured by hand while snorkeling at night in water up to 7 m depth in coral reefs of Gorgona National Park. (2°56′–3°02′N, 78°10′–78°13′W). This 9 km long and 2.5 km wide island with a total protected area of 617 km^2^ (including surrounding waters) is located 56 km from the mainland town of Guapi in the southern Colombian Pacific coast. The island is surrounded by near-shore coral reefs where green turtles are found throughout the year. Samples were taken from 55 green turtles ranging from 42.7 to 77.6 cm minimum curved carapace length (mCCL, measured from the nuchal scute to the posterior notch at midline between the supracaudals; mean = 61.2±S.D. 8.2 cm) which were subsequently tagged in both front flippers with Inconel 1005-681S tags (National Band & Tag Co.), using standard techniques [23]. About of 2–3 mm of skin tissue was removed from the neck or shoulder of each animal with a sterile scalpel following standard methodology [24]. Skin samples were preserved in a 20% DMSO solution saturated with NaCl and processed in the Laboratory of Molecular Biology and Tissue Bank of the Colombian Alexander von Humboldt Biodiversity Research Institute (IAvH), in Cali, Colombia.

### DNA extraction, PCR amplification and sequencing

DNA was extracted from samples using a Qiagen DNeasy kit (Qiagen, Germantown MD, USA) and DNA concentration and quality was visualized by electrophoresis of 5 µl on a 0.8% agarose gel stained with EtBr. We amplified a 550 base pair fragment of the mtDNA control region using primers LTCM2 and HDCM2 [25]. For the polymerase chain reaction (PCR) we used 2 µl of template DNA in 25 µl reaction volumes containing 50 ng of genomic DNA, 1 µl of *Taq* polymerase (Perkin-Elmer/Cetus, Norwalk, PA), 10 µM of each primer, 5 µM dNTPs (Deoxynucleotide Triphosphates), 25 µM MgCl_2_ and Buffer 10× (KCl 500 mM and 200 mM Tris-HCl, pH 8.4). The reaction consisted of 35 cycles of a thermocycler (PTC-100, MJ Research, Waltham MA, USA) applying 20 s at 94°C, 20 s at 53°C and 20 s at 72°C. The initial denaturing (94°C) and last extension (72°C) cycles were of 2 minutes each [26]. Reactions were verified in 1% agarose electrophoresis gel and successful PCR products were then purified using the Poly Ethylene Glycol [27] method (20% PEG 8000, 2.5 M NaCl) prior to sequencing in both directions in a Multichannel Capillary Electrophoresis sequencer (ABI 310 - 3100).

### MtDNA haplotype characterization and data analysis

The mtDNA sequences were edited and assembled using Chromas Pro v1.34 (Technelysium Pty Ltd, Tewantin QLD, Australia) and trimmed before alignment with CLUSTAL X v1.74 [28]. Sequences were aligned and compared to reference sequences in order to identify haplotypes. Each nucleotide change encountered in an individual sequence was considered a different haplotype. Haplotypes encountered in Gorgona National Park were compared with Pacific Ocean sea turtle haplotypes reported in the National Marine Fisheries Service - Southwest Fisheries Science Center (NMFS-SWFSC) Marine Turtle Research Program website (http://swfsc.noaa.gov/prd-turtles.aspx). In order to allow comparisons with published studies in other FG and nesting stocks in the Pacific basin sequences were truncated at an internal 384 bp segment used universally by others [10], [19], [29] (Table 1).

**Table 1:** mtDNA control region haplotype (384 bp) frequencies for Gorgona National Park foraging grounds, compared with other published East and central Pacific rookeries and foraging grounds.

				East and central Pacific rookeries				East and central Pacific foraging grounds		
				Mexico		Ecuador	Hawaii	Hawaii		Colombia
	Gorgona designation	std SWFSC haplotype nomenclature	GENBANK Accession numbers	Michoacan [30]	Revillagigedo [31]	Galapagos [31]	FFS [31]	total foraging [31]	strandings [31]	Gorgona
		CMP1			1		156	477	76	
		CMP2					34	82	16	
		CMP3			11		39	112	22	
	GPC1	CMP4	AY382323	82	23	95			1	46
		CMP5		34						
		CMP6			50			1		
		CMP7		2						
	GPC3	CMP8	AY382326	2						1
		CMP9			2					
		CMP10			1					
		CMP11			1					
		CMP12		3						
		CMP13			1					
	GPC2	CMP15	AY540063			3				1
	GPC4	CMP17	AY540065							1
		CMP20						1		
	GPC6	CMP21	AY540069							1
	GPC5	CMP22	AY540070							3
	GPC7	CMP97	FJ268479							2
**Total haplotypes**				5	8	2	3	5	4	7
**No. of animals**				123	90	98	229	673	115	55
**Nesting pop. size (nests.yr^−1^)**				1,395 [39]	90 [40]	1,650 [41]	400 [42]	N/A	N/A	N/A

A selection of haplotypes from Central, Eastern Pacific [30], [31] and Australasian rookery clades [19] as well as from regional foraging grounds in Japan (haplotypes CMJ12, CMJ3 GenBank acc no. AB472311, AB472302) [32], Palmyra Atoll (CMP109; acc. no. GU12196), and French Polynesia (CMPo1, CMPo3; acc. no. EF555564, EF555566) were included to help detect probable geographic origins for orphan haplotypes found at the Gorgona FG (GPC5, GPC6, GPC7).

A 10 bp insertion at position 355 in haplotypes E1, E2 and CMJ12 was coded as a single substitution for further analyses. Haplotype frequencies, Nei's [33] haplotype diversity (h) and nucleotide diversity (π) from the control region sequences were calculated using Arlequin v.3.11 [34]. The phylogenetic relationships between Gorgona and reference haplotypes data from Dethmers et al [19] were inferred from MEGA4 [35] using the Neighbor-Joining method [36] on the basis of genetic distances computed using the Tamura-Nei model and a Pacific loggerhead (*Caretta caretta*) sequence as the outgroup (GenBank acc. no. U22261). Genetic differentiation between the Gorgona foraging aggregation and eastern Pacific nesting and foraging aggregations, was quantified using 10,000 permutations for ϕST [37] under the Tamura-Nei model [38]. All computations were carried out by the program Arlequin v.3.11 [34].

Rookery contributions to the Gorgona green turtle aggregation were estimated using Bayesian Mixed Stock Analyses (MSA) as implemented in the many-to-many model described by Bolker et al. [7] using both weighted and unweighted applications. Source haplotype profiles for the MSA were taken from eastern (Michoacan, Revillagigedos and Galapagos) and central (Hawaii) Pacific rookeries [10], [30]. Source population size (as nests/yr) was used as prior information in the weighted model for Michoacan, Mexico [39], Galapagos, Ecuador [40], [41] and French Frigate Shoals, Hawaii USA [42]. Identifying potential origins for turtles with orphan haplotypes was attempted on the basis of sequence similarities using the phylogenetic relationships mentioned previously.

## Results

### Haplotype composition

**Figure 3:**
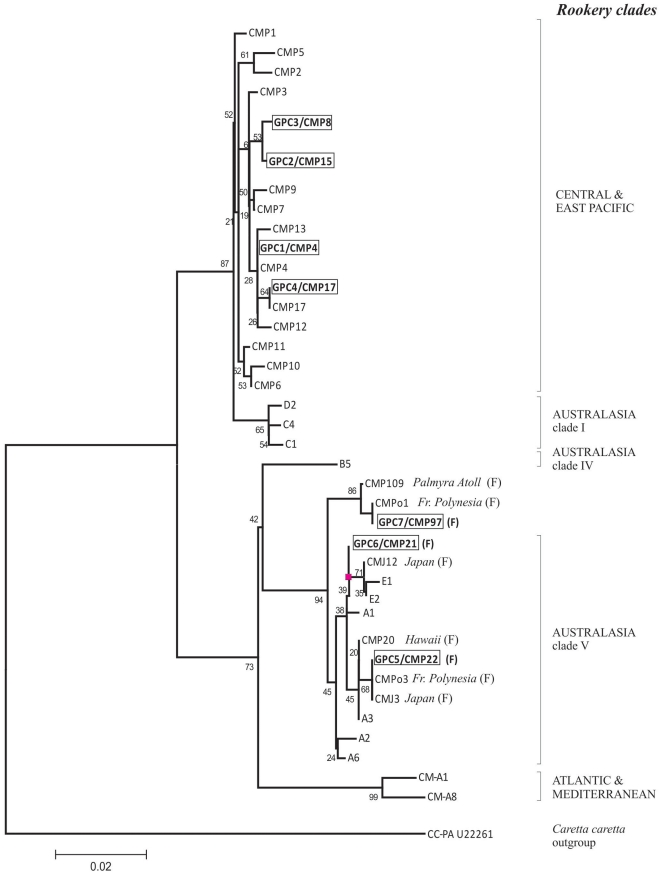
Phylogeny of representative green turtle mtDNA control region haplotypes. Presented using the Neighbor-Joining method [36] with branch lengths proportional to the sequence divergence indicated by the scale and the bootstrap values at each branch. A selection of haplotypes from central, eastern Pacific [30], [31] and Australasian rookery clades [19] as well as regional foraging grounds [32] were included to help detect probable geographic origins for orphan haplotypes found at the Gorgona FG (CMP22, CMP97). Haplotypes for green turtles found at the study site are in boxes and bold type. Red square indicates presence of 10 bp insertion. Haplotypes from foraging or bycatch are indicated by an “F”, all others are from nesting sites. The *Caretta caretta* haplotype (GenBank acc. No. U22261) was used as an outgroup.

Seven haplotypes (GPC1-7) based on 26 variable sites were identified from the 55 sequences of green turtles sampled at Gorgona (Table 1). All have been previously described [10], [19] although no nesting origin has been reported for haplotypes GPC5, GPC6 and GPC7 (CMP21, CMP22, and CMP97, respectively). The most common haplotype at Gorgona was GPC1 (CMP4) in 83% of the sampled population, which is also the most common at both of the major green turtle rookeries in the eastern Pacific: Galapagos, Ecuador [10] and Michoacan, Mexico [30]. Two of the minor haplotypes, both found in single individuals (GPC2 and GPC3; CMP15 and CMP18) have been reported from rookeries nesting in Michoacan and Galapagos, respectively [10], [30]. Haplotype GPC4 (CMP17), also found in a single individual, although belonging to the same central and eastern Pacific lineage as the previous sequences, has no reported rookery origin (Figure 3). The three remaining haplotypes (GPC5, GPC7, CMP22, CMP21, and CMP97, respectively), representing 11% of the total sample, probably stem from western Pacific rookeries as judged by their sequence similarities to lineages from that region (Figure 2). None of these sequences have been reported for any rookery in that or other regions, although CMP21 and CMP22 (4 and 5% of samples, respectively) are identical at the 384 bp reading frame to sequences reported for the species at foraging habitats in French Polynesia (CMPo3 and CMPo1) and Japan (CMJ3) [32]. The sequence for haplotype GPC6 (CMP21; from a single individual) is identical to that from haplotypes E2 [19] and CMJ12 [32] except for a 10 bp insertion at position 355 that these latter sequences have and that is absent in GPC6. Haplotype E2 was reported from two rookeries in Micronesia (Elato and Ngulu Atolls) [19].

### Genetic and morphological diversity

Although the haplotype diversity (h = 0.30±0.080) and the number of resolved haplotypes (NH = 7) at Gorgona FG falls within the range reported for other green turtle foraging aggregations (Atlantic h = 0.18–0.77, NH = 2–13; Hawaii h = 0.46, NH = 5; Table 2), the nucleotide diversity (π) 0.011±0.006 is the second highest recorded for the species at a FG.

**Table 2:** Comparison of mtDNA control region sequence diversities in the Gorgona green turtle FG with the species' published nesting and foraging aggregations, measured as haplotype diversity and nucleotide diversity.

Geographic site	No. of haplotypes	Haplotype diversity *(h)* ± s.e.	Nucleotide diversity (*π*) ± s.e.	Sample size (animals)	Source
***Nesting grounds***					
**ATLANTIC**					
Wider Caribbean					
Florida, USA	11	0.61±0.103	0.001±0.001	44	[25]
Quintana Roo, Mexico	7	0.82+0.058	0.006	20	[50]
Isla Aves, Venezuela	2	0.25±0.18	0.005	8	[50]
Surinam	15	0.25±0.141	0.0006	15	[50]
Tortuguero, Costa Rica	5	0.16±0.020	0.003±0.002	433	[49]
**PACIFIC**					
Australasia region	25	0.88±0.010	0.041±0.020	714	[19]
Michoacan, Mexico	5	0.48±0.040	0.003±0.002	123	[30]
Revillagigedo, Mexico	8	0.61±0.042	0.003±0.002	90	[31]
FFS Hawaii, USA	3	0.49±0.032	0.003±0.002	229	[31]
***Foraging grounds***					
**ATLANTIC**					
Almofala, Brasil	13	0.71±0.030	0.006±0.000	117	[16]
Ubatuba, Brasil	10	0.44±0.556	0.002±0.001	113	[16]
North Carolina, USA	12	0.72±0.030	0.005±0.003	106	[6]
Florida, USA	6	0.48±0.066	0.003±0.002	62	[63]
Bahamas	6	0.37±0.065	0.006±0.00	79	[25]
Barbados	8	0.77±0.029	0.010±0.005	60	[64]
Nicaragua	2	0.18±0.062	0.003±0.002	60	[65]
**PACIFIC**					
FFS Hawaii, USA	5	0.46±0.020	0.002±0.002	673	[31]
strandings FFS Hawaii, USA	4	0.51±0.044	0.003±0.002	115	[31]
Yaeyama Islands, Japan	11	0.842	0.024	142	[32]
Gorgona, Colombia	7	0.30±0.080	0.011±0.006	55	this study

The mean mCCL for the 55 sampled turtles was 61. 2±8.2 cm and ranged between 42.7 and 77.6 cm. On the basis of a classification for nesting female sizes of over 75 cm CCL for Galapagos turtles (converted from original SCL) [43], we assumed the presence of a total of 47 juveniles (<70 cm CCL), 7 sub adults (70–75 cm CCL) and one large juvenile of unspecified sex (>75 cm CCL) in our sample.

There were characteristic morphological differences amongst carapaces of individuals captured at the Gorgona FG. Carapace color for most of the sampled turtles (85%, n = 47) was greenish - black, which is commonly associated with the eastern Pacific green turtles [44], although the Revillagigedo nesters and foraging animals in San Diego Bay have more variable carapace shape and coloration [40], [45]–[47]. All of these turtles were associated with the common East Pacific haplotypes CMP4 and CMP8. Remaining sampled turtles (15%, n = 8) had variable carapace colorations different from green-black and included yellowish–brown, pale green and dark yellow (Figure 3), and were associated with haplotypes falling within phylogenetic clades of sequences from green turtles sampled in central (CMP15 and CMP17) and western Pacific (CMP21, CMP22 and CMP97) rookeries.

### Genetic differentiation

When comparing the Gorgona FG haplotype profiles with those from green turtle aggregations of central and eastern Pacific habitats (Table 1) both the exact test of differentiation (results not shown) and the ϕST values revealed the Gorgona turtles as being statistically distinct (ϕST 0.12–0.58) (all P<0.01; Table 3). Notably, the level of differentiation was lowest when comparing against eastern Pacific aggregations and the lowest genetic distance value was found when comparing with the Galapagos rookery.

**Table 3:** Genetic distances (ϕST, Tamura-Nei model) between the Gorgona FG green turtle aggregation and other Pacific nesting and feeding aggregations.

Type of habitat	Population	Tamura-Nei ϕ_st_	Source	P value
Rookeries	Michoacan	0.144	[30]	<.001
	Revillagigedo	0.281	[31]	<.001
	Galapagos	0.116		<.01
	Hawaii FFS	0.497		<.001
Foraging grounds	Hawaii	0.575	[31]	<.001
	Hawaii (strandings)	0.429		<.001

### Mixed Stock Analysis

**Figure 4:**
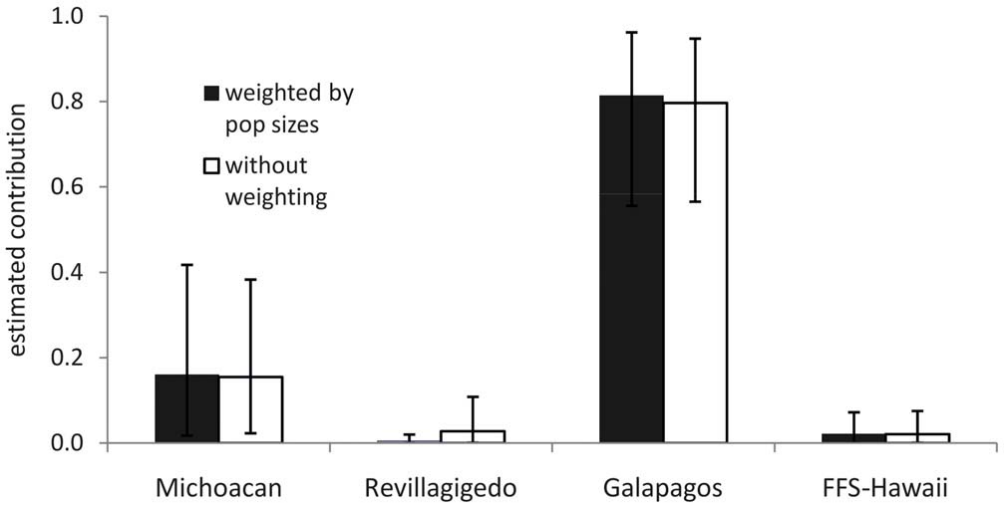
Bayesian mixed stock estimates of contributions to the Gorgona foraging ground by central and eastern Pacific rookeries, after excluding putative western Pacific “orphan” haplotypes. Dark bars represent results using source population sizes (nests/yr) information; light bars without source population size. Source haplotype profiles used were obtained for Michoacan and Revillagigedo (Mexico), Galapagos (Ecuador) and French-Frigate Shoals Hawaii (USA). Error bars represent 97.5% and 2.5% percentile intervals.

To conduct the mixed stock analysis, we removed the orphan haplotypes (6 of the total of 55 individuals sampled), all of which consisted of sequences phylogenetically associated with the Central-Eastern (CMP17) and Western Pacific (CMP21, CMP22 and CMP97) lineages. With the remaining set of samples, the mixed stock analysis (MSA) identified and quantified contributions to our study site from genetically characterized Eastern (Michoacan, Revillagigedo, Galapagos) and Central Pacific (Hawaii) rookeries (Figure 4). Weighting the analysis by abundances of the source populations, the major contributing rookeries from these regions appeared to be Galapagos 80% (55–96%, 95% confidence interval) and Michoacan 15% (2–38%, 95% confidence interval), while Revillagigedo and Hawaii appeared to not contribute significantly (<2%). The proportions of contribution by rookery remained the same when the MSA was run with uninformative priors.

## Discussion

The results of this study provide genetic evidence to support previous speculation that green turtle stock at Gorgona FG is composed of individuals recruited from multiple nesting sites in the Pacific [14], [15]. These findings contribute important information to updating the Colombian Sea Turtle Action Plan, and for strengthening protection of the Eastern Pacific Tropical Marine Corridor (CMAR). They also highlight the value of inferences that can be drawn by considering the foraging strategies, population size-class composition and residence of turtles in non-nesting habitats for the implementation of national and regional wildlife policy planning.

### Genetic composition and diversity of Gorgona foraging aggregation

The haplotype (h) and nucleotide (π) diversities indicate the Gorgona FG contains turtles that probably originate from a broad geographic range, since this parameter (π) increases with sequence divergence. The seven haplotypes identified in Gorgona FG suggests that this MPA represents a critical site potentially used as developmental and/or stopover habitat by a number of green turtle stocks from both sides of the Pacific Ocean basin. The western Pacific haplotypes may be rare, but could be present at some of the under-sampled eastern Pacific nesting sites or others that have not yet been surveyed. By maintaining the health and richness of Gorgona's marine habitats these FG can help sustain a large number of turtles from different breeding origins, considering that over 700 individuals have been tagged since 2001 (Amorocho, unpublished data).

The genetic diversity estimated for the Gorgona foraging grounds is within the ranges for green turtle feeding aggregations in other FG of the Pacific [10], [19], [48] and Atlantic sites [6], [8], [25], [49], [50] as shown in Table 2. The overall haplotype (*h* = 0.300) and nucleotide (*π* = 080) diversities were relatively low, due to the dominance of haplotype CMP4, present in 46 (83%) of the 55 turtles. Despite the limited number of sampled individuals and comparatively low number of haplotypes (seven), high nucleotide differences were observed within the Gorgona aggregation. The *π* value was also high compared to other localities in the Atlantic such as Brazil (Ubatuba 0.002; Almofala 0.006), Tortuguero in Costa Rica (0.003) and other foraging sites in the wider Caribbean (0.000–0.005) (Table 2). The relatively high haplotype and nucleotide diversity found in our study indicate that Gorgona is an important place for green turtles in order to maintain population and genetic variability in the eastern Pacific, contributing to diversity of Regional Management Units (RMUs) [51].

### Role of sea surface currents

**Figure 5:**
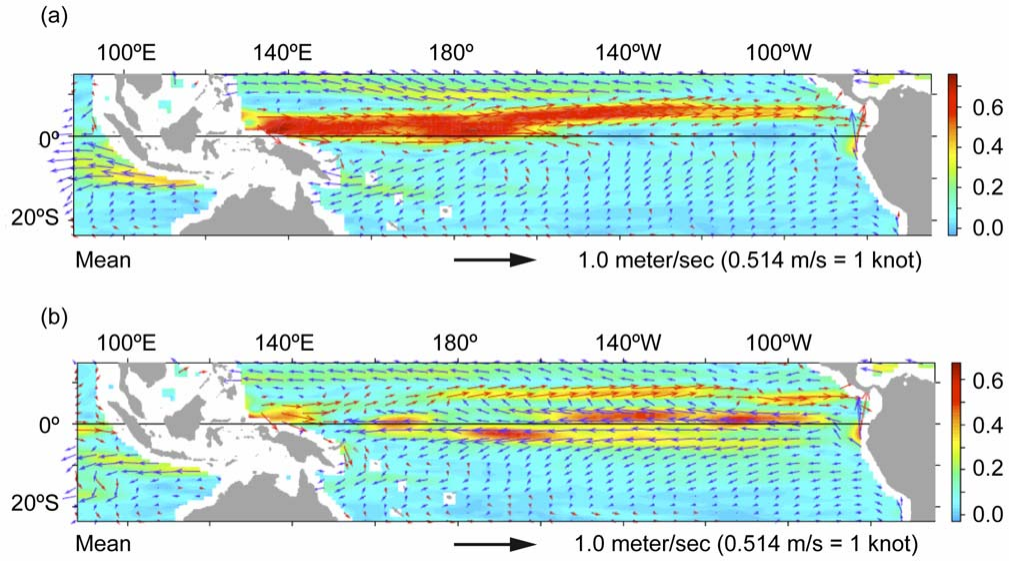
Mean surface ocean currents in the Pacific region 15°N–24°S 100°E–60°W from NOAA Ocean Surface Current Analyses- Real Time program. (a) Averages for July–December 1997 (during a very intense El Niño) indicating the strong and extensive eastward flowing North Equatorial Counter-current (NEECC) as a major feature of the current pattern. (b) Averages for July–December 2000 (common, non-El Niño conditions) showing the presence of the NECC as weaker and reduced compared to the westward flowing Equatorial current. Red overlay vector arrows indicate eastward flows; blue arrows indicate westward flows. Colored contour plots indicate current speed (meters per second) according to scale on the right. Map and information downloaded from http://www.oscar.noaa.gov/datadisplay/.

**Figure 6:**
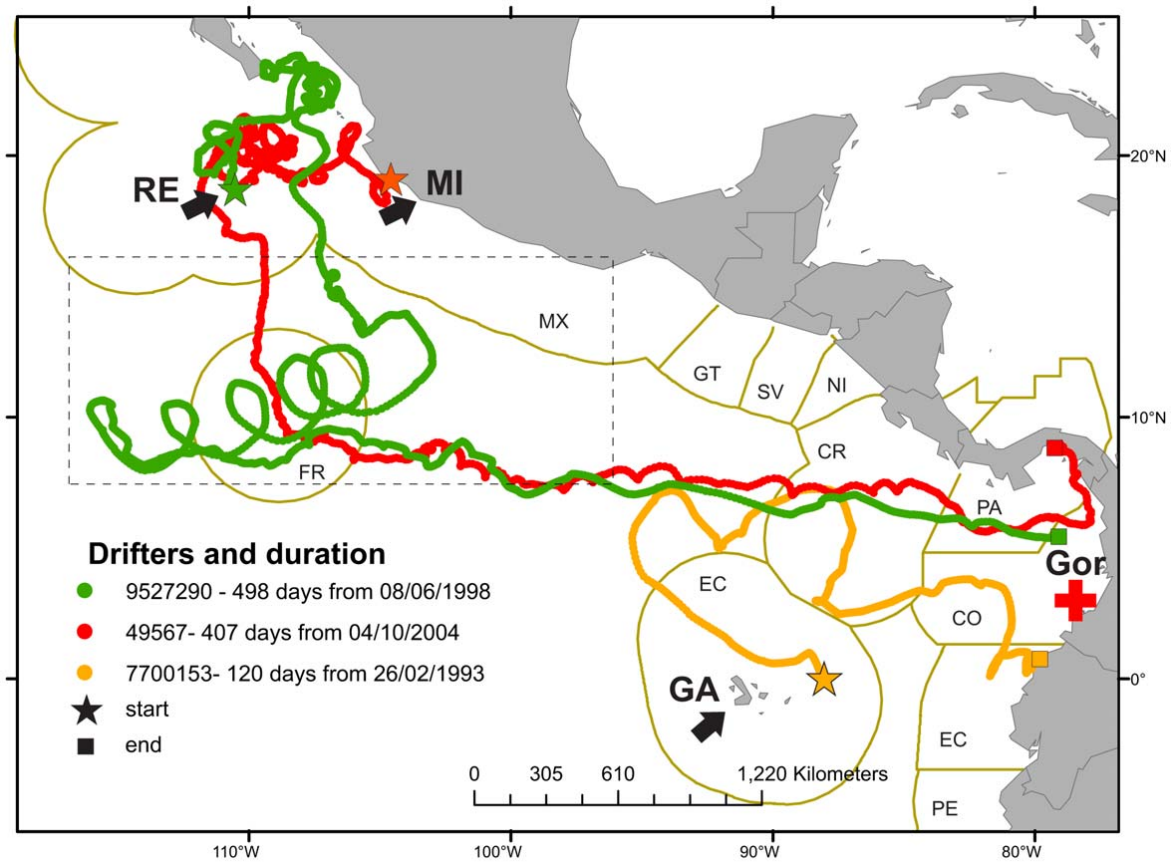
Satellite-tracked drifter buoy trajectories demonstrating potential ocean current pathways linking green turtle breeding areas in the Eastern Pacific with the Gorgona foraging site. Tracks from three drifters deployed near Eastern Pacific breeding ground heading towards the vicinity of the Gorgona study site (red cross). RE = Revillagigedo Islands, Mexico; MI = Michoacan, Mexico; GA = Galapagos Islands, Ecuador; Gor = Gorgona Island, Colombia. Rectangle in broken lines highlight area with frequent eddies provoking recurrent looped tracks with increased speed (about 2X average) but longer entrainment within the current system. Countries' EEZ boundaries are indicated with two-letter abbreviations. Drifter data from NOAA/AOML Global Lagrangian Drifter Data (http://www.aoml.noaa.gov/envids/gld/krig/parttrk_id_temporal.php).

**Figure 7:**
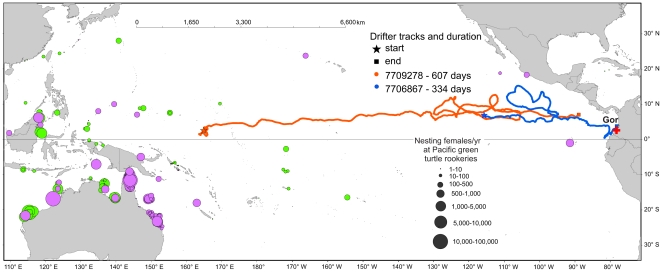
Satellite-tracked drifter buoy trajectories demonstrating potential ocean current pathways linking green turtle breeding areas in the Western Pacific with the Gorgona foraging site. Two drifter tracks initiating on the eastern edge of Western Pacific green turtle habitats leading to areas near the Gorgona study site (red cross). Total duration indicated in the figures. Green turtle rookery locations and abundances derived from IOSEA Marine Turtle Mapping System (http://stort.unep-wcmc.org/imaps/indturtles) and the Marine Turtle Database maintained by C. J. Limpus at Queensland Parks and Wildlife Service, Australia. Purple circles indicate Pacific basin breeding colonies that have been genetically characterized (see Table 2); green circles show populations with no genetic studies. Drifter data from NOAA/AOML Global Lagrangian Drifter Data (http://www.aoml.noaa.gov/envids/gld/krig/parttrk_id_temporal.php).

To complement the MSA results and to help explain genetic links between the major regional green turtle rookeries and the Gorgona FG, we explored plausible regional dispersal routes by ocean currents (Figure 5), using the trajectories of satellite-tracked Lagrangian drifter buoys (http://www.aoml.noaa.gov/). The location of Revillagigedo and Michoacan rookeries places them at a junction between the southerly moving California Current and the North Equatorial Current flowing due West [52]. Drifter data is scarce in this general area, but tracks were recovered that are consistent with drift scenarios for small juvenile green turtles from these rookeries to the Gorgona area. These tracks follow different paths, but terminate in a region very close to Gorgona (Figure 6), as did a drifter deployed East of Galapagos, consistent with the finding of Michoacan and Galapagos haplotypes in the Gorgona FG. Thus, although we did not find genetic contribution from Revillagigedo, there is the potential for it to be present. No drifter deployed in the vicinity of FFS-Hawaii reached the general Gorgona area and oceanic current transport of buoy drifters from Western Pacific habitats to Gorgona appeared to be more likely than from Hawaii, despite being much further away. Transport through the North Pacific Drift was discounted as being too cold for green turtles [53]. Two tracks were found terminating relatively close to Gorgona that suggest that passive transport from Western Pacific habitats would take around 2 years, but is possible (Figure 7), providing a potential route into the Gorgona aggregation for animals from these distant populations. It must be noted that current systems in the Eastern Pacific are very variable [52], as is the direction and scope of potential transport by the North Equatorial Countercurrent, so drift of turtles to Gorgona from distant regions may be sporadic. This preliminary analysis suggests that currents could be responsible for the transport of green turtle juveniles towards Gorgona, but more in-depth studies are needed to further determine how dispersal is influenced by currents.

### Dispersal, recruitment and migratory behavior of Gorgona turtles

Our findings reinforce what has been suggested in other sea turtle studies; that the recruitment into FG is influenced by oceanic mixing of individuals during their multi-year pelagic stage [6], [54]. Equatorial currents may be an important vehicle for dispersal of green turtle post-hatchlings from western to eastern Pacific regions. After arriving at neritic areas such as Gorgona, turtles likely spend some time recovering from the transoceanic phase and storing resources to continue travel to further developmental and mating grounds. Our results indicate that most (estimated 55–96%) of Gorgona green turtles come from nesting beaches of the Galapagos, with potential contribution (estimated 2–38%) from rookeries in Michoacan State (Mexico). This suggests the existence of marine gateways, or paths connecting Michoacan State and Galapagos nesting rookeries with the FG of Gorgona and Galapagos [55], [56] in the south-eastern Pacific. These three sites are part of the same green turtle management unit at a regional scale [51]. The results of our Gorgona MSA also provide new information related to potential linkages between eastern Pacific FG and distant nesting populations from FFS-Hawaii rookery (Figure 1, Table 2). The presence of western Pacific haplotypes has also been noted in green turtles foraging around the Galapagos Islands [56], suggesting further connectivity between these regional foraging populations. More comprehensive work including all the key Pacific rookeries and new genetic markers should provide more accurate estimates of the stock composition and connectivity between the regional nesting and foraging aggregations [7], [57]–[59].

However, the Gorgona turtles with western Pacific haplotypes may merely be drifters transported by currents to the eastern Pacific that do not return to their natal beaches to breed. Gorgona and other habitats along the eastern Pacific would therefore act as genetic ‘sinks’ for these individuals. The Lagrangian drifter data show that it is possible for the eastward drift to occur over the vast distance to reach Gorgona, as has been shown for transatlantic movements of green turtle juveniles [3].

Some evidence of morphotype and size class similarities between Gorgona and Galapagos green turtles (from where a large proportion of the individuals at Gorgona FG originate) suggest migration or drifting between FG through the eastern Pacific pelagic and/or neritic habitats. Migratory paths for Galapagos post-nesting green turtles including, oceanic migration to Central America, residency within the Galapagos and travel into oceanic waters southwest of the Galapagos [55] are consistent with this suggestion. Consequently, a more comprehensive MSA is needed to determine if the CMP4 haplotype is present in other eastern Pacific FG or rookeries that have not yet been studied. The large error for the Michoacan and Galapagos contributions to the Gorgona MSA are probably due to the low haplotype diversity observed in this study and the sharing of haplotype CMP4 between Michoacan and Galapagos. Improvement in the accuracy of the MSA will require increased sample sizes, the inclusion of additional molecular markers and an expanded baseline of potential regional sources. Satellite or GPS telemetry will contribute to better understanding the dispersal and migratory movements of green turtle juveniles after they leave Gorgona. The mapped routes of satellite tracked animals supported by flipper tagging data will be a useful tool in designing accurate plans for the species conservation management in the Pacific. In order to fully identify the nesting origin of reported haplotypes in Gorgona, more breeding beaches need to be surveyed along the eastern tropical Pacific coast and molecular assignments developed to determine the percentage and contribution of haplotypes from each Pacific site to Gorgona's green turtle aggregation. Surveys must also target females that occasionally breed at Palmeras beach in southwest Gorgona Island [60] and at the northern Colombian Pacific beach of La Cuevita, to test the possibility that these nesting sites are the origin of orphan haplotypes. Green turtles foraging in Gorgona could also be recruited from nesting beaches of coastal Machalilla National Park [61] in Ecuador or the Galapagos islands [62].

### Conservation implications

Haplotypes identified so far have provided valuable information to the Colombian environmental authorities for the establishment of multinational strategies such as the Eastern Tropical Pacific Marine Corridor (CMAR). This is a marine conservation regional initiative carry out by the governments of Ecuador, Colombia, Panama and Costa Rica to protect MPAs of Galapagos (Ecuador); Malpelo and Gorgona (Colombia); Coiba (Panama) and Cocos (Costa Rica). Acknowledging the presence of individuals mainly from Galapagos in Ecuador and Mexico demonstrates to the Colombian government the need to sign the Inter American Convention for Sea Turtle Protection (IAC). The accomplishment of these transnational agreements would help not only to ensure the survival of sea turtles in the Pacific Ocean but also the coordinated management of other marine trans-boundary resources [10]. The convergence at Gorgona FG of green turtles from distinct, distant rookeries and the role it plays providing shelter and food is important for conservation management in the eastern Pacific and for the linkage between MPAs and protected nesting beaches along the CMAR. However, the role of Gorgona and other eastern Pacific FG in the ecology of western Pacific green turtle populations still remains unclear.

Our genetic findings combined with ongoing mark – recapture studies and satellite tracking will be helpful for connecting MPAs in a broader scale for better implementation of regional sea turtle management plans. This study is the first step toward characterizing a FG in Colombia and together with further movement tracking studies will be relevant to elucidate linkages between nesting and FG for improvement of current and the design of new MPAs. In addition, more genetic surveys of green turtle nesting beaches are required and together with comprehensive MSA will help ensure that the ecological role of Gorgona and other marine and coastal protected areas along the eastern Pacific region are recognized.
